# The Fees of Institution Medical Officers

**Published:** 1914-11-14

**Authors:** 


### THE FEES OF INSTITUTION MEDICAL OFFICERS.

The institution medical officer has always been treated somewhat unfairly in respect of payment for the notification of infectious disease. A good instance of this was noted last week in reference to the position of the medical superintendents of tuberculosis institutions with regard to the payment for notifications under the Public Health (Tuberculosis) Regulations. As he has accepted a whole-time appointment, it is deemed to be unnecessary to pay him even the small fee awarded to the general practitioner for information given to the local sanitary authority, although that information has proved to be of the greatest value for the benefit of the public health. To avoid throwing a serious charge upon local authorities, the institution officer has, throughout the phases of tuberculosis notification, been called upon to bear the brunt of the new work without adequate reward.

This disability of the institution officer with regard to fees is already a serious and a growing one. It applies, or attempts are made to apply it, in the matter of fees for vaccination; such bodies as Boards of Guardians induce their medical officers to "compound" upon appointment for fees for the certification of the insane, and even to undertake to repay all law-court attendance and other fees received. But little can be said in favour of the practice of compounding such fees in estimating salaries; they should be paid in direct recognition of special professional ability or care and of the legal liability involved. The absurdity of the position as regards fees for *post-mortem* examinations and attendance at a Coroner's court is only too well known; in connection with an inquest concerning the death of a person who dies before reaching the hospital door fees are paid to the medical officer, whilst, should the patient survive admission by a few moments, the fees are refused. The skill required in the examination of the body and the ability, caution, and responsibility called for in giving evidence are the same under the two conditions, yet the position remains an anomaly still impervious to ridicule and common sense. It is to be feared that similar treatment of the institution medical officer may be expected when the certification of causes of death becomes the direct concern of the State.

The value of the notification of tuberculous disease depends upon the means exercised by the sanitary authorities and their officers to prevent the spread of infection. In the case of tuberculosis (as, indeed, in that of other infections) those means must consist especially of either (i) the removal of the patient from home, or (ii) the improvement of conditions at home, and (iii) disinfection applied in view of the special infective dangers. In the application and choice of these preventive means the tuberculosis dispensary and its medical officer are coming to bear an increasingly important part. The transference of the patient to an institution is a very important step from the point of view of the medical officer of health, and is generally only made known to him when he receives a notification of the admission, upon form C, from the institution medical officer. Similarly, without a notification, upon form D, of the patient's discharge from an institution, the medical officer of health may be entirely unaware of the return home, or even of the introduction into his district, of a patient suffering possibly from advanced disease. Many such patients are content to remain for long periods without medical attendance, in the absence of which re-notification or "following-up" may be evaded. The institution officer's notification is more, rather than less, valuable than that of the general practitioner, and it is unfortunate that the fact should not have been recognised in arranging the scale of notification fees.

